# Crystal structure of 1-{3-(4-methyl­phen­yl)-5-[(*E*)-2-phenyl­ethen­yl]-4,5-di­hydro-1*H*-pyrazol-1-yl}ethan-1-one

**DOI:** 10.1107/S2056989015024792

**Published:** 2015-12-31

**Authors:** Farook Adam, Kanathur Smitha, Sharath Poojary Charishma, Seranthimata Samshuddin, Nadiah Ameram

**Affiliations:** a School of Chemical Sciences, Universiti Sains Malaysia, 11800, Pulau Pinang, Malaysia; b Department of PG Studies in Chemistry, Alva’s College, Moodbidri, Karnataka 574 227, India

**Keywords:** crystal structure, synthesis, pyrazoline, pharmacological properties

## Abstract

The title compound, C_20_H_20_N_2_O, was studied as a part of our work on pyrazoline derivatives. It represents a *trans*-isomer. The central pyrazoline ring adopts an envelope conformation with the asymmetric C atom having the largest deviation of 0.107 (1) Å from the mean plane. It forms dihedral angles of 6.2 (1) and 86.4 (1)° with the adjacent *p*-tolyl and styrene groups, respectively. In the crystal, C—H⋯O inter­actions link mol­ecules into infinite chains along the *c* axis.

## Related literature   

For background to pyrazoles, see: Samshuddin *et al.* (2012 ▸); Wiley *et al.* (1958 ▸); Sarojini *et al.* (2010 ▸); Lu *et al.*(1999 ▸). For crystal structures of pyrazoline-derived chalcones, see: Jasinski *et al.* (2012 ▸); Baktır *et al.* (2011 ▸). For stability of the temperature controller used for the data collection, see: Cosier & Glazer (1986 ▸).
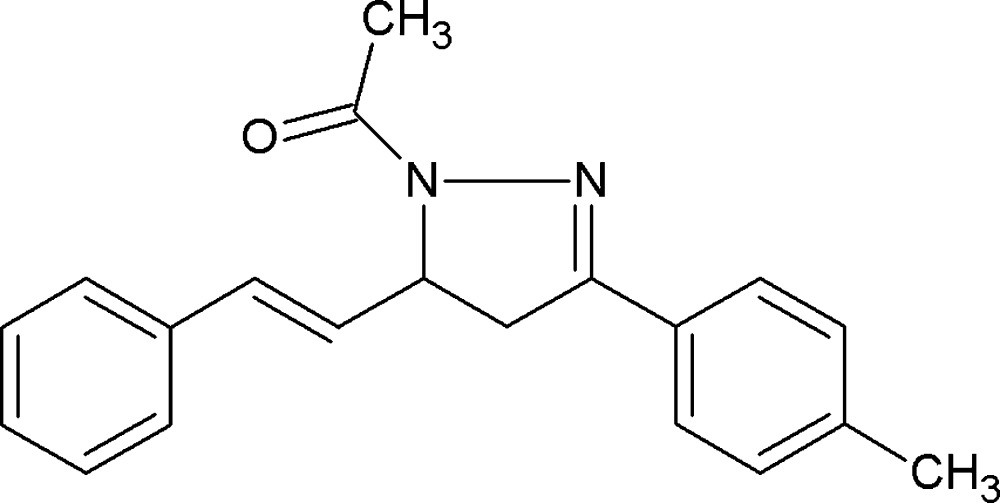


## Experimental   

### Crystal data  

C_20_H_20_N_2_O
*M* *_r_* = 304.38
Orthorhombic, 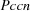

*a* = 19.872 (2) Å
*b* = 20.304 (2) Å
*c* = 8.2924 (8) Å
*V* = 3345.9 (6) Å^3^
*Z* = 8
Mo *K*α radiation
μ = 0.08 mm^−1^
*T* = 100 K
0.38 × 0.31 × 0.17 mm

### Data collection  

Bruker APEX DUO CCD area-detector diffractometer
Absorption correction: multi-scan (*SADABS*; Bruker, 2009 ▸) *T*_min_ = 0.914, *T*_max_ = 0.960
60332 measured reflections
5482 independent reflections
4234 reflections with *I* > 2σ(*I*)
*R*_int_ = 0.041

### Refinement  

*R*[*F* ^2^ > 2σ(*F* ^2^)] = 0.052
*wR*(*F* ^2^) = 0.146
*S* = 1.04
5482 reflections
210 parameters
H-atom parameters constrained
Δρ_max_ = 0.37 e Å^−3^
Δρ_min_ = −0.27 e Å^−3^

### 

Data collection: *APEX2* (Bruker, 2009 ▸); cell refinement: *SAINT* (Bruker, 2009 ▸); data reduction: *SAINT*; program(s) used to solve structure: *SHELXS97* (Sheldrick, 2008 ▸); program(s) used to refine structure: *SHELXL2014* (Sheldrick, 2008 ▸); molecular graphics: *SHELXTL* (Sheldrick, 2008 ▸); software used to prepare material for publication: *PLATON* (Spek, 2009 ▸) and *publCIF* (Westrip, 2010 ▸).

## Supplementary Material

Crystal structure: contains datablock(s) I, New_Global_Publ_Block. DOI: 10.1107/S2056989015024792/ld2138sup1.cif

Structure factors: contains datablock(s) I. DOI: 10.1107/S2056989015024792/ld2138Isup3.hkl

Supporting information file. DOI: 10.1107/S2056989015024792/ld2138Isup3.cml

**Fig. 1.**
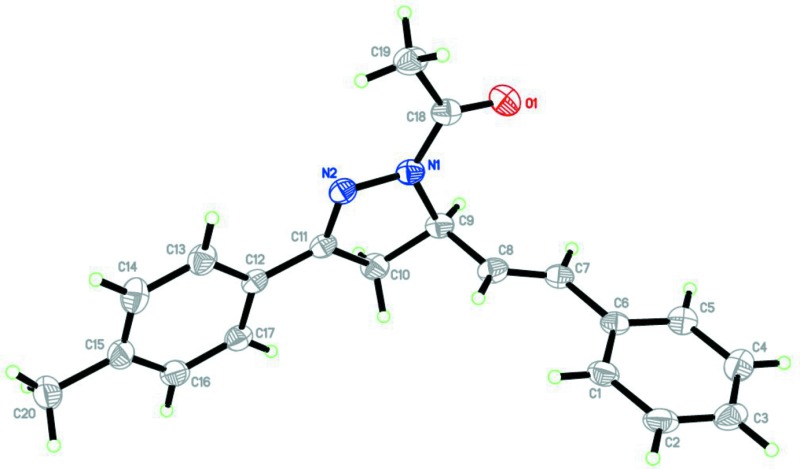
The molecular structure of title compound (I) with atom labels and 50% probability displacement ellipsoids.

**Fig. 2.**
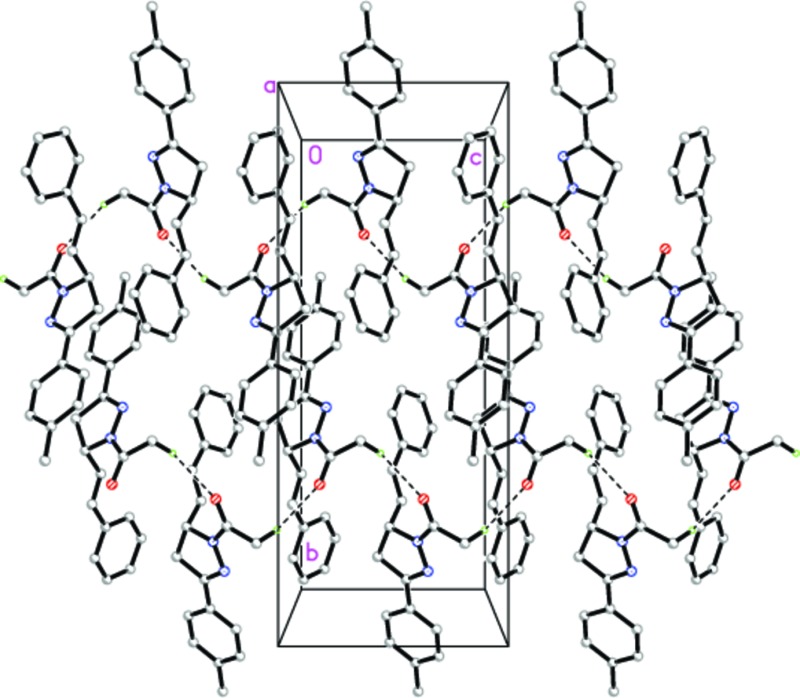
The crystal packing of title compound (I) viewed along the a-axis.

. DOI: 10.1107/S2056989015024792/ld2138fig1.tif
The mol­ecular structure of title compound (I) with atom labels and 50% probability displacement ellipsoids.

a . DOI: 10.1107/S2056989015024792/ld2138fig2.tif
The crystal packing of title compound (I) viewed along the *a*-axis.

CCDC reference: 1444202

Additional supporting information:  crystallographic information; 3D view; checkCIF report

## Figures and Tables

**Table 1:** Hydrogen-bond geometry (Å, °)

*D*—H⋯*A*	*D*—H	H⋯*A*	*D*⋯*A*	*D*—H⋯*A*
C19—H19*C*⋯O1^i^	0.98	2.51	3.4416 (18)	158

Symmetry code: (i) 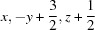.

## supplementary crystallographic information

### S1. Introduction

Pyrazoline derivatives exhibit numerous pharmacological activities including anti­oxidant, anti­amoebic, anti-inflammatory, analgesic, anti­microbial, anti depressant and anti­cancer activities (Sarojini *et al.*, 2010; Samshuddin *et al.*, 2012). Many 1,3,5-tri­aryl-2-pyrazolines were used as scintillation solutes (Wiley *et al.*, 1958) and as fluorescent agents (Lu *et al.*, 1999). The crystal structures of some pyrazolines containing *N*-alkyl chain *viz*., 3,5-bis­(4-fluoro­phenyl)-4,5-di­hydro-1*H*-pyrazole-1-carbaldehyde (Baktir *et al.*, 2011), 3,5-bis­(4-fluoro­phenyl)-4,5-di­hydro-1*H*-pyrazole-1-carboxamide and 3,5-bis­(4-fluoro­phenyl)-4,5-di­hydro-1*H*-pyrazole-1-carbo­thio­amide (Jasinski *et al.*, 2012) had been reported. In view of the importance of pyrazolines, the title compound (I) is prepared and its crystal structure is reported.

### S2. Experimental

A mixture of (2*E*,4*E*)-1-(4-methyl­phenyl)-5-phenyl­penta-2,4-dien-1-one (2.48 g, 0.01 mol) and hydrazine hydrate (1 ml) in 30 ml acetic acid was refluxed for 6 h·The reaction mixture was cooled and poured into 100 ml ice-cold water. The precipitate was collected by filtration and purified by recrystallization from ethanol.

### S2.1. Synthesis and crystallization

Single crystals were grown from ethanol by slow evaporation method. m.p.: 386–390 K. Yield: 71%.

### S2.2. Refinement

All H atoms were placed in calculated positions and refined with riding model [*U*_iso_ (H) = 1.2 × *U*_eq_(C methyl­ene or methine) or 1.5 × *U*_eq_ (C methyl), C—H = 0.95 Å, 0.98 Å, 0.99 Å and 1.00 Å]. A rotating group model (AFIX 137) is applied to methyl groups.

### S3. related literature

For background of pyrazoles, see: Samshuddin *et al.* (2012); Wiley *et al.* (1958); Sarojini *et al.* (2010); Lu *et al.* (1999). For crystal structures of pyrazoline derived chalcone, see: Jasinski *et al.* (2012); Baktir *et al.* (2011). For stability of the temperature controller used for data collection, see: Cosier & Glazer (1986). For ring conformations, see: Cremer & Pople (1975).

### S4. Results and discussion

The asymmetric unit of (I) consists of a single crystallographic independent molecule as shown in Fig. 1. The C6/C7/C8/C9 carbon chains adopts a *trans* configuration with respect to C7—C8 double bond. The pyrazoline ring (N1/N2/C9/C10/C11) adopts an envelope conformation on atom C9 [Q2 = 0.1772 (12) Å and φ2 = 317.3 (4)°] with maximum deviation of 0.107 (1) Å from its mean plane. The *p*-tolyl ring and styrene group make dihedral angles of 6.19 (7)° and 86.39 (7)° with central pyrazoline ring. In crystal, molecules are connected by weak C—H···O hydrogen bond into one-dimensional chains (Fig. 2), propagating along crystallographic *c*-axis.

### Crystal data

**Table:**

C_20_H_20_N_2_O	*D*_x_ = 1.208 Mg m^−^^3^
*M**_r_* = 304.38	Mo *K*α radiation, λ = 0.71073 Å
Orthorhombic, *P**c**c**n*	Cell parameters from 9875 reflections
*a* = 19.872 (2) Å	θ = 2.8–31.0°
*b* = 20.304 (2) Å	µ = 0.08 mm^−^^1^
*c* = 8.2924 (8) Å	*T* = 100 K
*V* = 3345.9 (6) Å^3^	Block, colourless
*Z* = 8	0.38 × 0.31 × 0.17 mm
*F*(000) = 1296	

### Data collection

**Table:**

Bruker APEX DUO CCD area-detector diffractometer	5482 independent reflections
Radiation source: fine-focus sealed tube	4234 reflections with *I* > 2σ(*I*)
Graphite monochromator	*R*_int_ = 0.041
φ and ω scans	θ_max_ = 31.4°, θ_min_ = 1.4°
Absorption correction: multi-scan (*SADABS*; Bruker, 2009)	*h* = −28→28
*T*_min_ = 0.914, *T*_max_ = 0.960	*k* = −29→29
60332 measured reflections	*l* = −12→12

### Refinement

**Table:**

Refinement on *F*^2^	0 restraints
Least-squares matrix: full	Hydrogen site location: inferred from neighbouring sites
*R*[*F*^2^ > 2σ(*F*^2^)] = 0.052	H-atom parameters constrained
*wR*(*F*^2^) = 0.146	*w* = 1/[σ^2^(*F*_o_^2^) + (0.0673*P*)^2^ + 1.4451*P*] where *P* = (*F*_o_^2^ + 2*F*_c_^2^)/3
*S* = 1.04	(Δ/σ)_max_ = 0.001
5482 reflections	Δρ_max_ = 0.37 e Å^−^^3^
210 parameters	Δρ_min_ = −0.27 e Å^−^^3^

### Special details

**Table:**

Geometry. All e.s.d.'s (except the e.s.d. in the dihedral angle between two l.s. planes) are estimated using the full covariance matrix. The cell e.s.d.'s are taken into account individually in the estimation of e.s.d.'s in distances, angles and torsion angles; correlations between e.s.d.'s in cell parameters are only used when they are defined by crystal symmetry. An approximate (isotropic) treatment of cell e.s.d.'s is used for estimating e.s.d.'s involving l.s. planes.

### Fractional atomic coordinates and isotropic or equivalent isotropic displacement parameters (Å^2^)

**Table:**

	*x*	*y*	*z*	*U*_iso_*/*U*_eq_	
O1	−0.07312 (5)	0.73524 (5)	0.64575 (12)	0.0353 (2)	
N1	−0.00679 (5)	0.64670 (5)	0.61909 (12)	0.0251 (2)	
N2	0.01613 (5)	0.58545 (5)	0.66989 (12)	0.0240 (2)	
C1	0.18906 (6)	0.81809 (7)	0.65242 (17)	0.0309 (3)	
H1A	0.1938	0.7741	0.6900	0.037*	
C2	0.23498 (6)	0.86562 (7)	0.70037 (18)	0.0347 (3)	
H2A	0.2707	0.8541	0.7712	0.042*	
C3	0.22917 (7)	0.92989 (7)	0.64565 (16)	0.0339 (3)	
H3A	0.2610	0.9622	0.6780	0.041*	
C4	0.17664 (7)	0.94670 (7)	0.54333 (16)	0.0332 (3)	
H4A	0.1724	0.9907	0.5055	0.040*	
C5	0.13015 (7)	0.89918 (7)	0.49601 (15)	0.0285 (3)	
H5A	0.0942	0.9112	0.4266	0.034*	
C6	0.13563 (6)	0.83413 (6)	0.54917 (14)	0.0244 (2)	
C7	0.08568 (6)	0.78511 (6)	0.49662 (14)	0.0248 (2)	
H7A	0.0522	0.7998	0.4232	0.030*	
C8	0.08281 (6)	0.72257 (7)	0.54150 (15)	0.0275 (2)	
H8A	0.1151	0.7079	0.6180	0.033*	
C9	0.03274 (6)	0.67307 (6)	0.48142 (14)	0.0258 (2)	
H9A	0.0029	0.6922	0.3964	0.031*	
C10	0.06773 (7)	0.61000 (6)	0.42244 (15)	0.0292 (3)	
H10A	0.0456	0.5922	0.3247	0.035*	
H10B	0.1159	0.6179	0.3993	0.035*	
C11	0.05888 (6)	0.56455 (6)	0.56448 (14)	0.0232 (2)	
C12	0.09411 (6)	0.50166 (6)	0.58275 (14)	0.0234 (2)	
C13	0.08359 (7)	0.46040 (7)	0.71578 (16)	0.0311 (3)	
H13A	0.0527	0.4731	0.7975	0.037*	
C14	0.11770 (8)	0.40148 (7)	0.72904 (18)	0.0360 (3)	
H14A	0.1093	0.3739	0.8194	0.043*	
C15	0.16411 (7)	0.38152 (6)	0.61306 (18)	0.0315 (3)	
C16	0.17496 (7)	0.42273 (7)	0.48225 (17)	0.0317 (3)	
H16A	0.2068	0.4104	0.4023	0.038*	
C17	0.14018 (6)	0.48161 (6)	0.46596 (16)	0.0288 (3)	
H17A	0.1479	0.5086	0.3741	0.035*	
C18	−0.05635 (6)	0.68098 (6)	0.69553 (15)	0.0276 (2)	
C19	−0.08727 (7)	0.64978 (7)	0.84270 (17)	0.0339 (3)	
H19A	−0.0718	0.6041	0.8519	0.051*	
H19B	−0.1364	0.6505	0.8330	0.051*	
H19C	−0.0737	0.6744	0.9390	0.051*	
C20	0.20029 (8)	0.31682 (7)	0.6276 (2)	0.0421 (4)	
H20A	0.1884	0.2959	0.7303	0.063*	
H20B	0.2490	0.3243	0.6237	0.063*	
H20C	0.1871	0.2880	0.5383	0.063*	

### Atomic displacement parameters (Å^2^)

**Table:**

	*U*^11^	*U*^22^	*U*^33^	*U*^12^	*U*^13^	*U*^23^
O1	0.0345 (5)	0.0360 (5)	0.0353 (5)	0.0065 (4)	−0.0006 (4)	0.0001 (4)
N1	0.0237 (5)	0.0295 (5)	0.0222 (5)	−0.0003 (4)	0.0008 (4)	0.0024 (4)
N2	0.0230 (4)	0.0269 (5)	0.0220 (4)	−0.0028 (4)	−0.0009 (4)	0.0010 (4)
C1	0.0213 (5)	0.0345 (6)	0.0368 (7)	0.0047 (5)	−0.0022 (5)	−0.0026 (5)
C2	0.0192 (5)	0.0454 (8)	0.0395 (7)	0.0026 (5)	−0.0021 (5)	−0.0081 (6)
C3	0.0270 (6)	0.0434 (7)	0.0313 (6)	−0.0069 (5)	0.0075 (5)	−0.0094 (5)
C4	0.0396 (7)	0.0344 (7)	0.0255 (6)	−0.0059 (5)	0.0057 (5)	−0.0008 (5)
C5	0.0307 (6)	0.0338 (6)	0.0211 (5)	0.0004 (5)	0.0010 (4)	0.0007 (5)
C6	0.0203 (5)	0.0321 (6)	0.0207 (5)	0.0016 (4)	0.0032 (4)	−0.0014 (4)
C7	0.0212 (5)	0.0323 (6)	0.0210 (5)	0.0016 (4)	−0.0009 (4)	0.0014 (4)
C8	0.0224 (5)	0.0358 (6)	0.0244 (5)	−0.0011 (5)	−0.0027 (4)	0.0053 (5)
C9	0.0241 (5)	0.0320 (6)	0.0214 (5)	−0.0003 (4)	0.0001 (4)	0.0049 (4)
C10	0.0304 (6)	0.0334 (6)	0.0237 (5)	0.0011 (5)	0.0055 (5)	0.0045 (5)
C11	0.0209 (5)	0.0277 (5)	0.0212 (5)	−0.0043 (4)	−0.0013 (4)	0.0012 (4)
C12	0.0215 (5)	0.0255 (5)	0.0233 (5)	−0.0052 (4)	−0.0011 (4)	0.0002 (4)
C13	0.0363 (7)	0.0303 (6)	0.0267 (6)	−0.0032 (5)	0.0050 (5)	0.0017 (5)
C14	0.0461 (8)	0.0291 (6)	0.0327 (7)	−0.0024 (6)	0.0013 (6)	0.0054 (5)
C15	0.0290 (6)	0.0248 (6)	0.0406 (7)	−0.0038 (5)	−0.0060 (5)	−0.0015 (5)
C16	0.0260 (6)	0.0313 (6)	0.0378 (7)	−0.0027 (5)	0.0031 (5)	−0.0045 (5)
C17	0.0266 (6)	0.0305 (6)	0.0293 (6)	−0.0039 (5)	0.0040 (5)	0.0010 (5)
C18	0.0245 (5)	0.0341 (6)	0.0242 (5)	−0.0021 (5)	−0.0011 (4)	−0.0041 (5)
C19	0.0323 (6)	0.0402 (7)	0.0291 (6)	−0.0032 (5)	0.0081 (5)	−0.0042 (5)
C20	0.0385 (8)	0.0285 (6)	0.0593 (10)	0.0013 (6)	−0.0065 (7)	0.0007 (6)

### Geometric parameters (Å, º)

**Table:**

O1—C18	1.2228 (16)	C10—C11	1.5066 (17)
N1—C18	1.3625 (16)	C10—H10A	0.9900
N1—N2	1.3898 (14)	C10—H10B	0.9900
N1—C9	1.4855 (15)	C11—C12	1.4642 (17)
N2—C11	1.2907 (15)	C12—C17	1.3934 (17)
C1—C2	1.3865 (19)	C12—C13	1.4008 (17)
C1—C6	1.4023 (17)	C13—C14	1.3795 (19)
C1—H1A	0.9500	C13—H13A	0.9500
C2—C3	1.386 (2)	C14—C15	1.393 (2)
C2—H2A	0.9500	C14—H14A	0.9500
C3—C4	1.388 (2)	C15—C16	1.387 (2)
C3—H3A	0.9500	C15—C20	1.5025 (19)
C4—C5	1.3923 (19)	C16—C17	1.3876 (19)
C4—H4A	0.9500	C16—H16A	0.9500
C5—C6	1.3967 (18)	C17—H17A	0.9500
C5—H5A	0.9500	C18—C19	1.5061 (18)
C6—C7	1.4717 (17)	C19—H19A	0.9800
C7—C8	1.3244 (17)	C19—H19B	0.9800
C7—H7A	0.9500	C19—H19C	0.9800
C8—C9	1.4995 (17)	C20—H20A	0.9800
C8—H8A	0.9500	C20—H20B	0.9800
C9—C10	1.5370 (18)	C20—H20C	0.9800
C9—H9A	1.0000		
			
C18—N1—N2	123.57 (10)	C9—C10—H10B	111.4
C18—N1—C9	123.76 (10)	H10A—C10—H10B	109.2
N2—N1—C9	112.47 (9)	N2—C11—C12	122.10 (11)
C11—N2—N1	107.73 (10)	N2—C11—C10	113.89 (11)
C2—C1—C6	120.78 (13)	C12—C11—C10	124.00 (10)
C2—C1—H1A	119.6	C17—C12—C13	118.08 (12)
C6—C1—H1A	119.6	C17—C12—C11	119.80 (11)
C3—C2—C1	120.43 (13)	C13—C12—C11	122.11 (11)
C3—C2—H2A	119.8	C14—C13—C12	120.53 (13)
C1—C2—H2A	119.8	C14—C13—H13A	119.7
C2—C3—C4	119.62 (13)	C12—C13—H13A	119.7
C2—C3—H3A	120.2	C13—C14—C15	121.52 (13)
C4—C3—H3A	120.2	C13—C14—H14A	119.2
C3—C4—C5	120.07 (13)	C15—C14—H14A	119.2
C3—C4—H4A	120.0	C16—C15—C14	117.87 (12)
C5—C4—H4A	120.0	C16—C15—C20	121.06 (13)
C4—C5—C6	120.97 (12)	C14—C15—C20	121.06 (13)
C4—C5—H5A	119.5	C15—C16—C17	121.24 (12)
C6—C5—H5A	119.5	C15—C16—H16A	119.4
C5—C6—C1	118.13 (12)	C17—C16—H16A	119.4
C5—C6—C7	119.57 (11)	C16—C17—C12	120.75 (12)
C1—C6—C7	122.29 (12)	C16—C17—H17A	119.6
C8—C7—C6	126.46 (11)	C12—C17—H17A	119.6
C8—C7—H7A	116.8	O1—C18—N1	120.02 (12)
C6—C7—H7A	116.8	O1—C18—C19	122.77 (12)
C7—C8—C9	125.30 (11)	N1—C18—C19	117.19 (12)
C7—C8—H8A	117.3	C18—C19—H19A	109.5
C9—C8—H8A	117.3	C18—C19—H19B	109.5
N1—C9—C8	109.70 (10)	H19A—C19—H19B	109.5
N1—C9—C10	100.58 (9)	C18—C19—H19C	109.5
C8—C9—C10	111.35 (10)	H19A—C19—H19C	109.5
N1—C9—H9A	111.6	H19B—C19—H19C	109.5
C8—C9—H9A	111.6	C15—C20—H20A	109.5
C10—C9—H9A	111.6	C15—C20—H20B	109.5
C11—C10—C9	102.04 (10)	H20A—C20—H20B	109.5
C11—C10—H10A	111.4	C15—C20—H20C	109.5
C9—C10—H10A	111.4	H20A—C20—H20C	109.5
C11—C10—H10B	111.4	H20B—C20—H20C	109.5
			
C18—N1—N2—C11	174.87 (11)	N1—N2—C11—C10	−2.35 (14)
C9—N1—N2—C11	−10.15 (13)	C9—C10—C11—N2	12.88 (14)
C6—C1—C2—C3	−0.6 (2)	C9—C10—C11—C12	−168.09 (11)
C1—C2—C3—C4	0.6 (2)	N2—C11—C12—C17	−179.52 (11)
C2—C3—C4—C5	0.0 (2)	C10—C11—C12—C17	1.53 (18)
C3—C4—C5—C6	−0.45 (19)	N2—C11—C12—C13	−0.05 (18)
C4—C5—C6—C1	0.41 (18)	C10—C11—C12—C13	−179.00 (12)
C4—C5—C6—C7	−179.89 (11)	C17—C12—C13—C14	−0.50 (19)
C2—C1—C6—C5	0.11 (19)	C11—C12—C13—C14	−179.97 (12)
C2—C1—C6—C7	−179.58 (12)	C12—C13—C14—C15	1.0 (2)
C5—C6—C7—C8	−177.26 (13)	C13—C14—C15—C16	−0.3 (2)
C1—C6—C7—C8	2.4 (2)	C13—C14—C15—C20	−179.17 (13)
C6—C7—C8—C9	−177.71 (11)	C14—C15—C16—C17	−0.8 (2)
C18—N1—C9—C8	74.84 (14)	C20—C15—C16—C17	178.07 (13)
N2—N1—C9—C8	−100.12 (12)	C15—C16—C17—C12	1.3 (2)
C18—N1—C9—C10	−167.76 (11)	C13—C12—C17—C16	−0.58 (18)
N2—N1—C9—C10	17.27 (12)	C11—C12—C17—C16	178.90 (11)
C7—C8—C9—N1	−120.45 (14)	N2—N1—C18—O1	178.64 (11)
C7—C8—C9—C10	129.11 (13)	C9—N1—C18—O1	4.22 (18)
N1—C9—C10—C11	−16.55 (11)	N2—N1—C18—C19	−0.05 (17)
C8—C9—C10—C11	99.63 (11)	C9—N1—C18—C19	−174.47 (11)
N1—N2—C11—C12	178.60 (10)		

### Hydrogen-bond geometry (Å, º)

**Table:**

*D*—H···*A*	*D*—H	H···*A*	*D*···*A*	*D*—H···*A*
C19—H19*C*···O1^i^	0.98	2.51	3.4416 (18)	158

Symmetry code: (i) *x*, −*y*+3/2, *z*+1/2.
