# Supplementary figures and images for: Crystal structure of 1-{3-(4-methyl­phen­yl)-5-[(E)-2-phenyl­ethen­yl]-4,5-di­hydro-1H-pyrazol-1-yl}ethan-1-one
Source: Acta Crystallogr E Crystallogr Commun. 2015 Dec 31;71(Pt 12):o1095–6. doi: 10.1107/S2056989015024792 (PMC4719997; doi:10.1107/S2056989015024792)

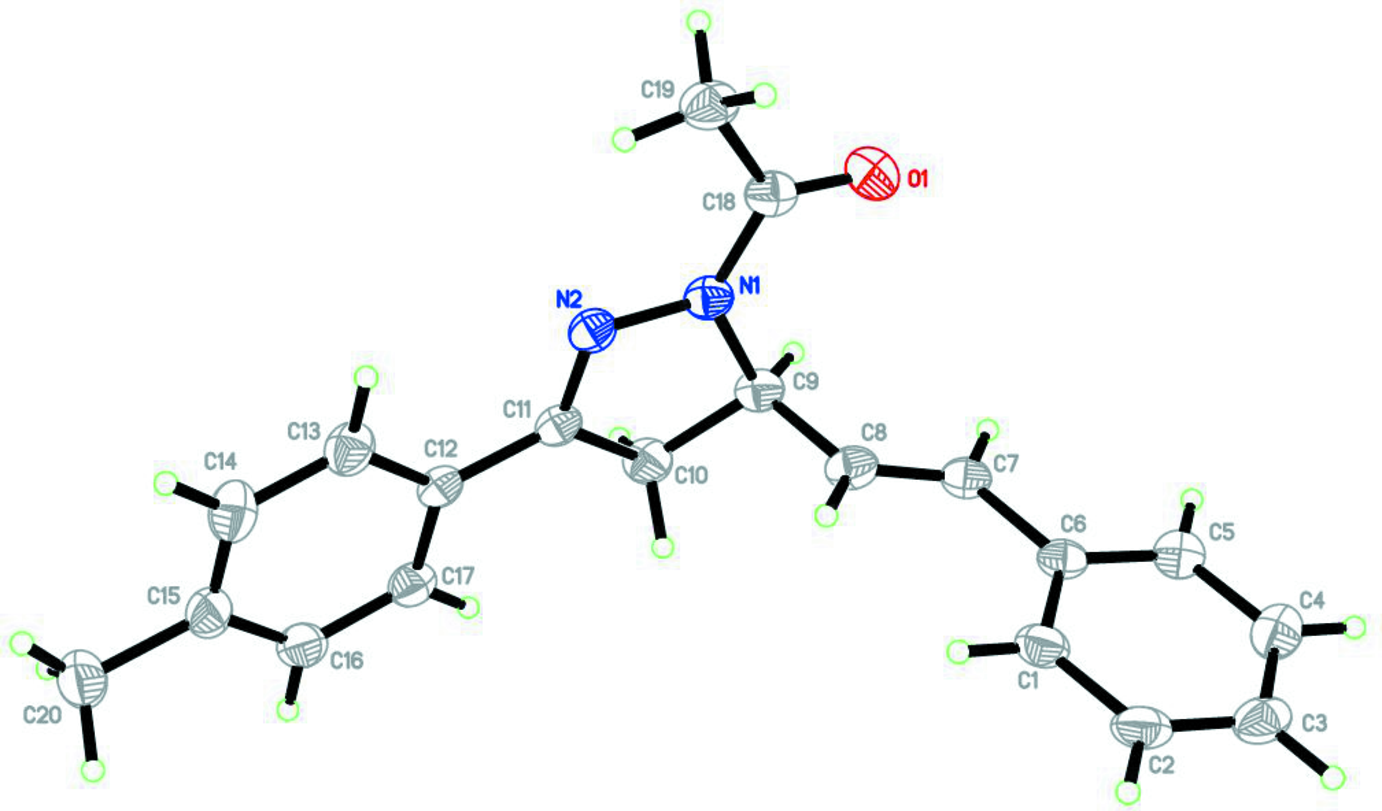

Supplement: Supplementary file 4 [file e-71-o1095-fig1.tif]

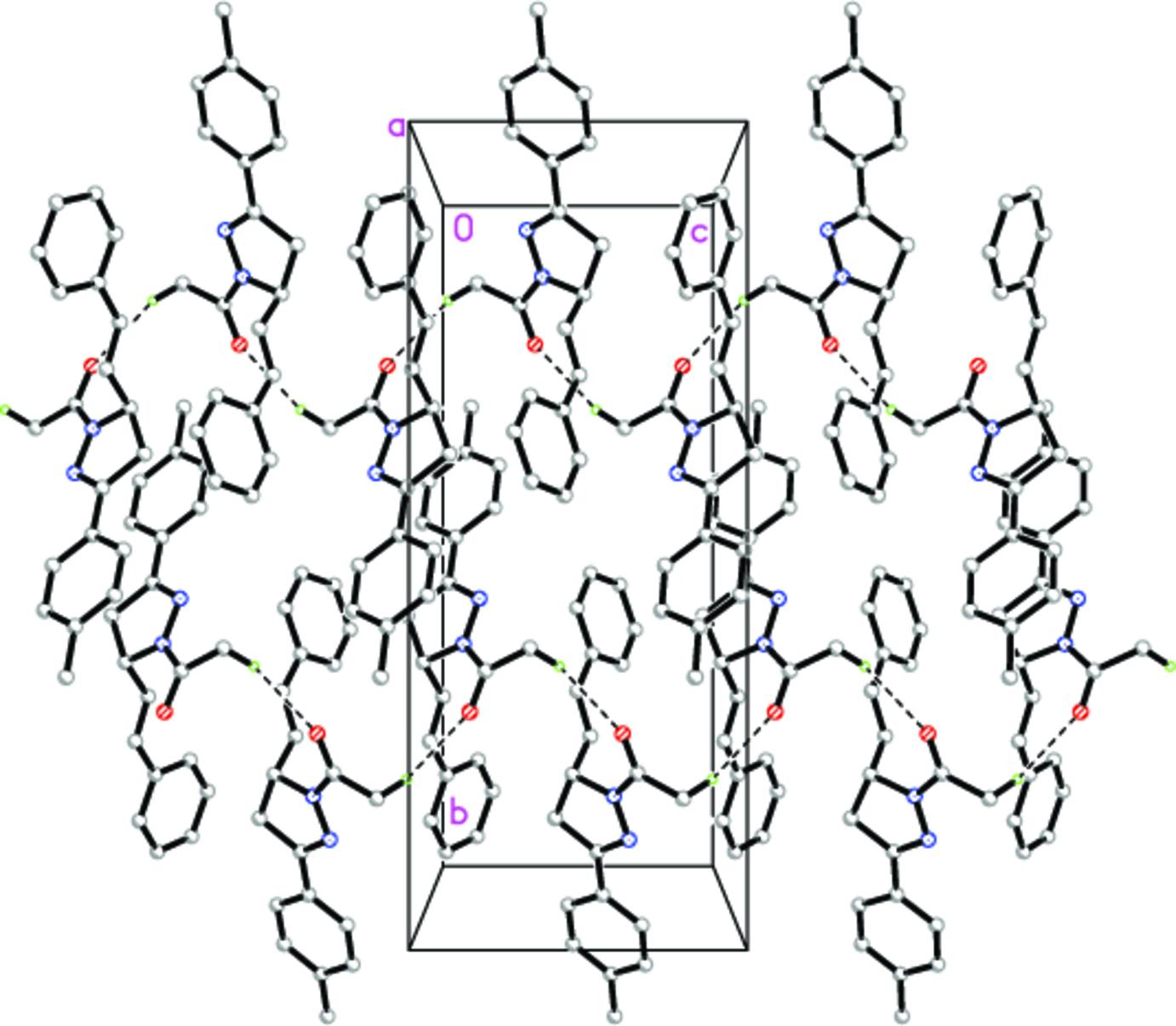

Supplement: Supplementary file 5 [file e-71-o1095-fig2.tif]
